# Mesenchymal Stem Cells in Synovial Fluid Increase After Meniscus Injury

**DOI:** 10.1007/s11999-013-3418-4

**Published:** 2013-12-13

**Authors:** Yu Matsukura, Takeshi Muneta, Kunikazu Tsuji, Hideyuki Koga, Ichiro Sekiya

**Affiliations:** 1 Department of Joint Surgery and Sports Medicine, Graduate School, Tokyo Medical and Dental University, Tokyo, Japan; 2 Department of Cartilage Regeneration, Graduate School, Tokyo Medical and Dental University, Tokyo, Japan; 3 Center for Stem Cell and Regenerative Medicine, Tokyo Medical and Dental University, 1-5-45 Yushima, Bunkyo-ku, Tokyo, 113-8519 Japan

## Abstract

**Background:**

Although relatively uncommon, spontaneous healing from a meniscus injury has been observed even within the avascular area. This may be the result of the existence of mesenchymal stem cells in synovial fluid.

**Questions/purposes:**

The purpose of this study was to investigate whether mesenchymal stem cells existed in the synovial fluid of the knee after meniscus injury.

**Methods:**

Synovial fluid was obtained from the knees of 22 patients with meniscus injury just before meniscus surgery and from 8 volunteers who had no history of knee injury. The cellular fraction of the synovial fluid was cultured for 14 days followed by analysis for multilineage potential and presentation of surface antigens characteristic of mesenchymal stem cells. Colony-forming efficiency and proliferation potential were also compared between the two groups.

**Results:**

Cells with characteristics of mesenchymal stem cells were observed in the synovial fluid of injured knees to a much greater degree than in uninjured knees. The colony-forming cells derived from the synovial fluid of the knee with meniscus injury had multipotentiality and surface epitopes identical to mesenchymal stem cells. The average number of colony formation, obtained from 1 mL of synovial fluid, in meniscus-injured knees was 250, higher than that from healthy volunteers, which was 0.5 (p < 0.001). Total colony number per synovial fluid volume was positively correlated with the postinjury period (r = 0.77, p < 0.001).

**Conclusions:**

Mesenchymal stem cells were found to exist in synovial fluid from knees after meniscus injury. Mesenchymal stem cells were present in higher numbers in synovial fluid with meniscus injury than in normal knees. Total colony number per synovial fluid volume was positively correlated with the postinjury period.

**Clinical Relevance:**

Our current human study and previous animal studies suggest the possibility that mesenchymal stem cells in synovial fluid increase after meniscus injury contributing to spontaneous meniscus healing.

## Introduction

The meniscus plays an important role in knee function and mechanics [24]. Meniscal injuries are a common and important source of knee dysfunction [13]. Meniscal repair is usually considered for the outer third of the meniscus because a rich network of arborizing vessels within the peripheral capsular and synovial attachments supplies vascularization to the menisci [7]. The remaining two-thirds of the meniscus have a poor vascular supply and thus a limited ability to heal spontaneously. However, spontaneous healing can be observed at the avascular area even in clinical situations (although relatively uncommon) [25] and in animal studies [3, 4, 16]. One of the possible mechanisms to account for this may be ascribed to the existence of mesenchymal stem cells in synovial fluid.

Mesenchymal stem cells are defined as being derived from mesenchymal tissue and having the functional capacity to self-renew and generate a number of differentiated progeny [2]. These cells participate in tissue homoeostasis, remodeling, and repair by ensuring replacement of mature cells that are lost during the course of physiological turnover, senescence, injury, or disease [1]. There are increasing reports that mesenchymal stem cells can be isolated from various adult mesenchymal tissues including intraarticular components [14, 19, 20, 26]. We previously reported that the number of mesenchymal stem cells in synovial fluid from knees with anterior cruciate ligament (ACL) injury and osteoarthritis was greater than that from healthy knees [17, 22]. Furthermore, the gene profiles of mesenchymal stem cells from synovial fluid were much closer to that of synovium than to that of bone marrow [17, 22].

According to our studies concerning meniscus regeneration in rat and rabbit models, synovium-derived mesenchymal stem cells injected into the knee adhered to the lesion, differentiated into meniscal cells directly or produced trophic support factors, and enhanced meniscus healing and regeneration [8, 10]. In a clinical situation, meniscus injuries have the potential to heal spontaneously, although it depends on the type and location of the lesion [25], raising the possibility that when the meniscus is injured, mesenchymal stem cells mobilize into synovial fluid, increase in number, and function to promote meniscal healing. However, the degree to which these cells may or may not be present in the human knee after meniscus injury has not been determined.

In this study, we investigated whether mesenchymal stem cells existed in synovial fluid of knees with meniscus injury and whether the number of mesenchymal stem cells in synovial fluid increased after meniscal injury in vivo in the human knee.

## Materials and Methods

### Collection of Synovial Fluid

This study was approved by an institutional review board, and informed consent was obtained from all study subjects. Synovial fluid was obtained from the knees of 22 patients with meniscus injury after induction of anesthesia for arthroscopic procedures for suture or partial resection of the injured meniscus in the operating room. These procedures took place at a mean of 12 weeks after injury (range, 2–39 weeks). Synovial fluid was also obtained from 8 healthy volunteers as a control group. Patients with anterior cruciate injury, a severe cartilage defect (International Knee Documentation Committee Grade 2–4), and osteoarthritis (Kellgren-Lawrence Grade 2–4) were eliminated from the study. In the injury group, mean age was 29 (SD, ± 17) years and mean postinjury period was 3.0 (SD, ± 2.3) months. In the control group, mean age was 34 (SD, ± 6) years.

### Definition of Mesenchymal Stem Cells

For purposes of our analysis, we defined a mesenchymal stem cell as one that has three characteristics [4]. First, mesenchymal stem cells must be adhered to plastic culture dishes and colony-formed when maintained in standard culture conditions. Second, the cells must express CD73, CD90, and CD105 at high rates and not express CD34 and CD45. Finally, the cells must differentiate into chondrocytes, adipocytes, and calcified tissue using standard in vitro conditions.

### Cultures of Colony-forming Cells in Synovial Fluid

**Fig. 1:**
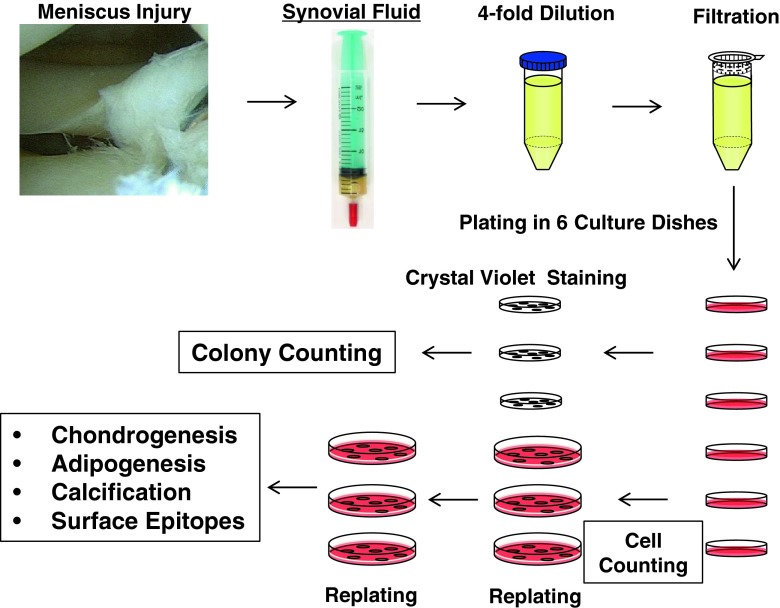
The study scheme is shown. Synovial fluid was obtained from knees with meniscus injury before surgery. Synovial fluid was diluted, filtered, and plated in six culture dishes. Fourteen days after initial plating, three dishes were stained with crystal violet and the number of colonies was counted. The remaining three dishes were harvested, number of cells counted, then replated and cultured for 14 days for further analyses.

Synovial fluid was diluted with three volumes of phosphate-buffered saline (PBS), filtered through a 70-μm nylon filter (Becton Dickinson, Franklin Lakes, NJ, USA) to remove debris, and plated in six culture dishes of 60-cm^2^ (Nalge Nunc International, Rochester, NY, USA) in complete culture medium: α-modified essential medium (α-MEM; Invitrogen, Carlsbad, CA, USA) containing 10% fetal bovine serum (Invitrogen), 100 U/mL penicillin, 100 μg/mL streptomycin, and 250 ng/mL amphotericin B (Invitrogen). The dishes were incubated at 37° C with 5% humidified CO_2_. After 24 hours, the nonadherent cells were washed out with PBS. Fourteen days after initial plating, three dishes were stained with 0.5% crystal violet (Wako, Osaka, Japan) in 4% paraformaldehyde for 10 minutes and the number of colonies was counted. Colonies less than 2 mm in diameter and faintly stained colonies were ignored. The other three dishes were harvested with 0.25% trypsin and 1 mM EDTA (Invitrogen) (Passage 0), and the number of isolated cells was counted. Then the cells were replated at 500 cells/cm^2^ in a 145-cm^2^ culture dish (Nalge Nunc International) and cultured for 14 days for further analyses (Fig. 1).

### Chondrogenesis

Two hundred thousand cells were placed in a 15-mL polypropylene tube (Becton Dickinson) and centrifuged at 450 x g for 10 minutes. The pellets were cultured at 37° C with 5% CO_2_ in 400 μL chondrogenesis medium that contained 1000 ng/mL BMP-7 (Stryker Biotech, Hopkinton, MA, USA) in high-glucose DMEM (Invitrogen) supplemented with 10 ng/mL transforming growth factor-β3 (R&D Systems, Minneapolis, MN, USA), 100 nM dexamethasone, 50 ng/mL ascorbate-2-phosphate, 40 μg/mL proline, 100 μg/mL pyruvate (Sigma-Aldrich, St Louis, MO, USA), and 50 mg/mL ITS + Premix (Becton Dickinson). The medium was replaced every 3 to 4 days for 21 days. For microscopy, the pellets were embedded in paraffin, cut into 5-μm sections, and stained with toluidine blue [23].

### Adipogenesis

One hundred cells were plated in 60-cm^2^ dishes and cultured in complete medium for 14 days. The medium was then switched to adipogenesis medium that consisted of complete medium supplemented with 100 nM dexamethasone, 0.5 mM isobutylmethylxanthine (Sigma-Aldrich), and 50 nM indomethacin (Wako) for an additional 21 days. The adipogenic cultures were fixed in 10% formalin and stained with fresh Oil Red-O (Sigma-Aldrich) solution [21].

### Calcification

One hundred cells were plated in 60-cm^2^ dishes and cultured in complete medium for 14 days. The medium was switched to calcification medium that consisted of complete medium supplemented with 1 nM dexamethasone (Sigma-Aldrich), 20 mM β-glycerol phosphate (Wako), and 50 μg/mL ascorbate-2-phosphate for an additional 21 days. These dishes were fixed in 10% formalin in PBS and stained with 40 mM alizarin red solution (pH 4.1; Sigma-Aldrich) [18].

### Epitope Profile

One million cells at Passage 2 were suspended in 500 μL PBS containing 20 μg/mL antibody. After incubation for 60 minutes at 4° C, the cells were washed with PBS and resuspended in 1 mL PBS for flow cytometric analysis. Fluorescein isothiocyanate (FITC), phycoerythrin-Cy7 (PE-Cy7), peridinin chlorophyll protein-Cy5.5 (PerCP-Cy5.5), or Allophycocyanin-H7 (APC-H7)-coupled antibodies against CD34, CD44, CD45, CD73, CD90, and CD105 (Becton Dickinson) were used. For isotype controls, FITC-, PE-Cy7-, PerCP-Cy5.5-, or APC-H7-coupled nonspecific mouse immunoglobulin G (IgG; Becton Dickinson) was substituted for the primary antibody. Cell fluorescence was evaluated by flow cytometry using a FACSVerse instrument (Becton Dickinson). The data were analyzed using FACSuite software (Becton Dickinson).

### Statistical Analysis

The Mann-Whitney U test was used for comparison of mesenchymal stem cell number and colony number between the control group and injury group. Probability values < 0.05 were considered significant. The correlation analysis was used for analyzing the relationship of the postinjury period and total colony number per volume.

## Results

### Analyses of Colony-forming Cells in Synovial Fluid

**Fig. 2A–B:**
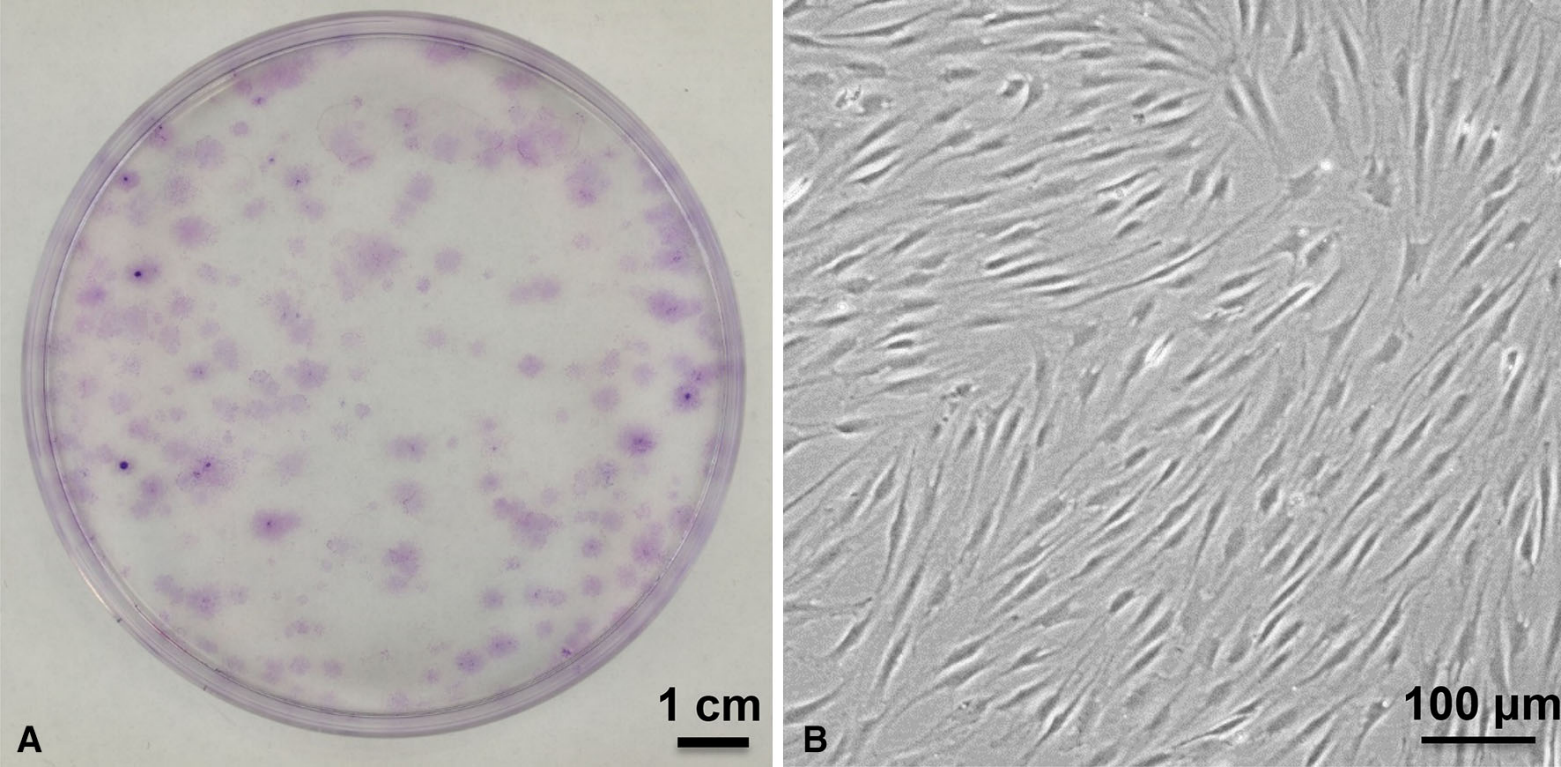
Colony-forming cells in synovial fluid derived from a meniscus injury to the knee are shown. (**A**) Representative cell colonies stained with crystal violet are shown. (**B**) Morphology of colony-forming cells is spindle-shaped (original magnification, ×100).

**Fig. 3A–C:**
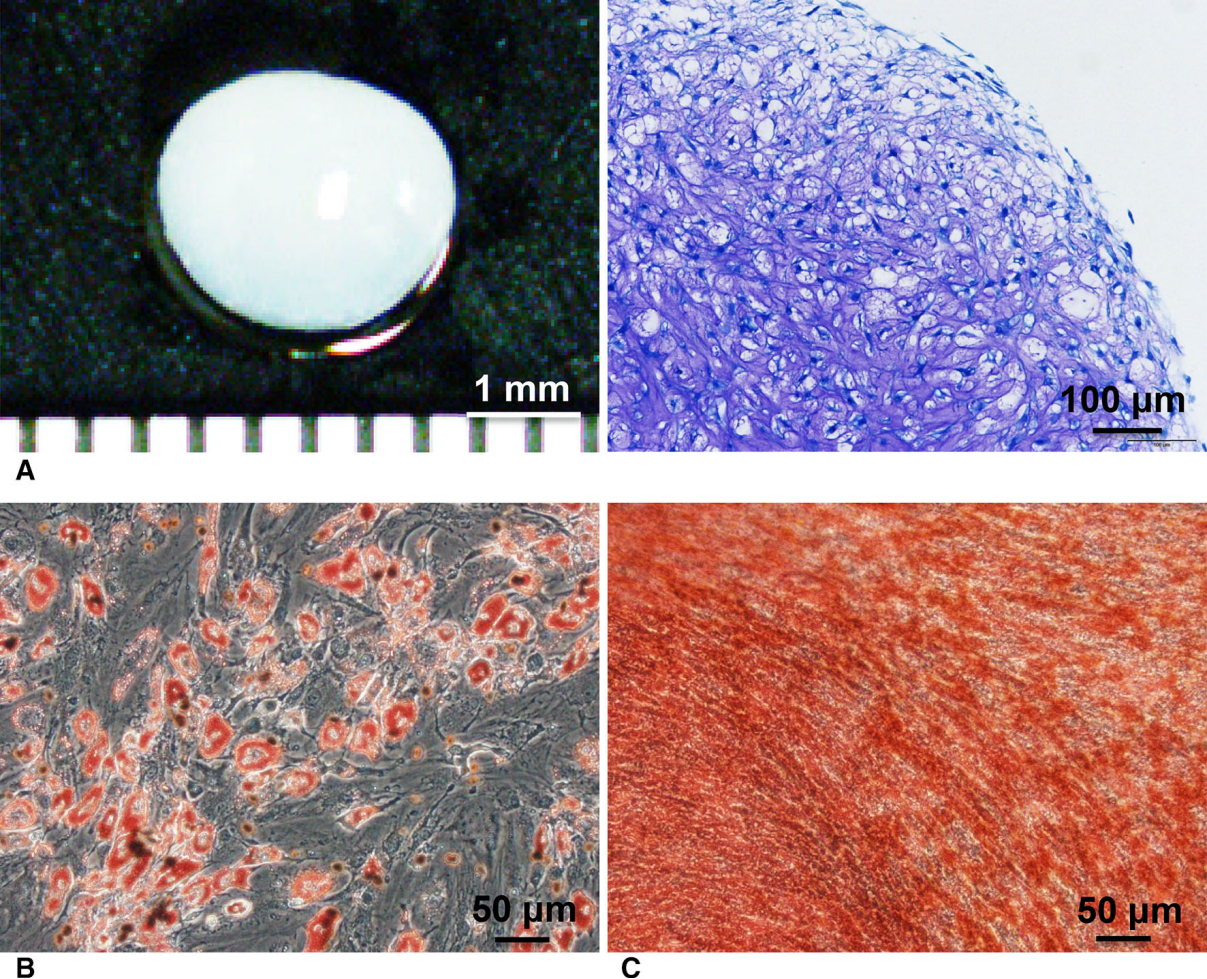
Colony-forming cells in synovial fluids have multipotentiality. (**A**) Cartilage and its histology are shown (Stain, toluidine blue; original magnification, ×100). (**B**) Adipocytes are shown (Stain, oil red-o; original magnification, ×200). (**C**) Calcified tissue is shown (Stain, alizarin red; original magnification, ×200).

**Fig. 4:**
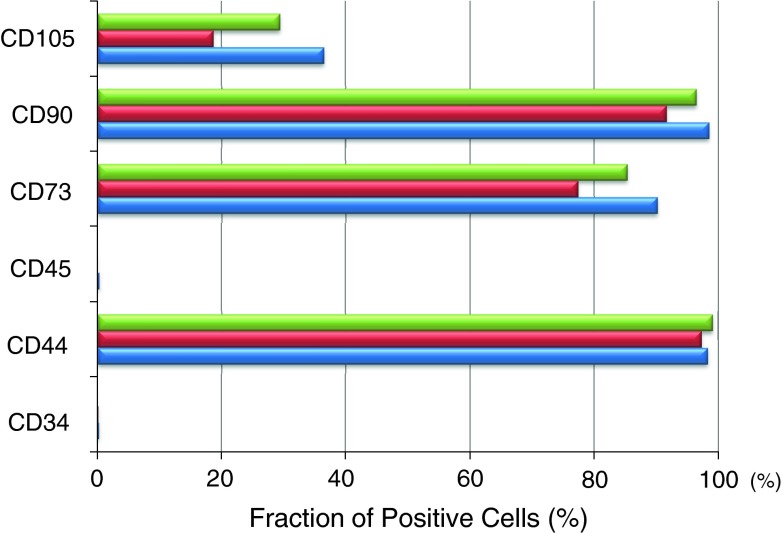
Surface epitopes of colony-forming cells in synovial fluids obtained from three donors are shown.

The colony-forming cells in synovial fluid obtained from the knees with meniscus injury had characteristics of mesenchymal stem cells. A large number of colonies composed of spindle-shaped cells was observed after 14 days’ culture of cell components in the synovial fluid of the knees with meniscus injury (Fig. 2). The colony-forming cells differentiated into chondrocytes, adipocyte, and calcified tissue when cultured in differentiation medium (Fig. 3). Three populations of colony-forming cells expressed CD44, CD73, and CD90 at high rates; CD105 at a moderate rate; and did not express CD34 or CD45 (Fig. 4). The colony-forming cells from uninjured knees could not be analyzed for multidifferentiation ability and surface epitopes because isolated cells were very few in number, as shown in the next section.

### Colony Formation of Synovial Fluid Mesenchymal Stem Cells in Meniscus Injury

**Fig. 5A–B:**
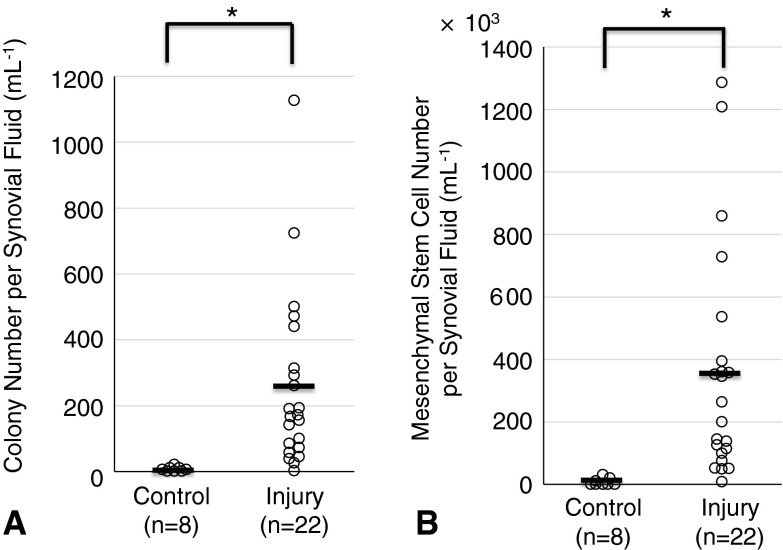
Quantification of mesenchymal stem cells derived from synovial fluid is shown. (**A**) Total colony number per volume (mL^−1^) of synovial fluid from healthy volunteers (control) and patients with meniscus injuries. Average values are shown as bars (n = 8 in control, n = 22 in injury, *p < 0.001 by Mann-Whitney U test). (**B**) Number of synovial fluid-derived mesenchymal stem cells per volume (mL^−1^) of synovial fluid (n = 8 in control, n = 22 in injury, *p < 0.001 by Mann-Whitney U test).

**Fig. 6:**
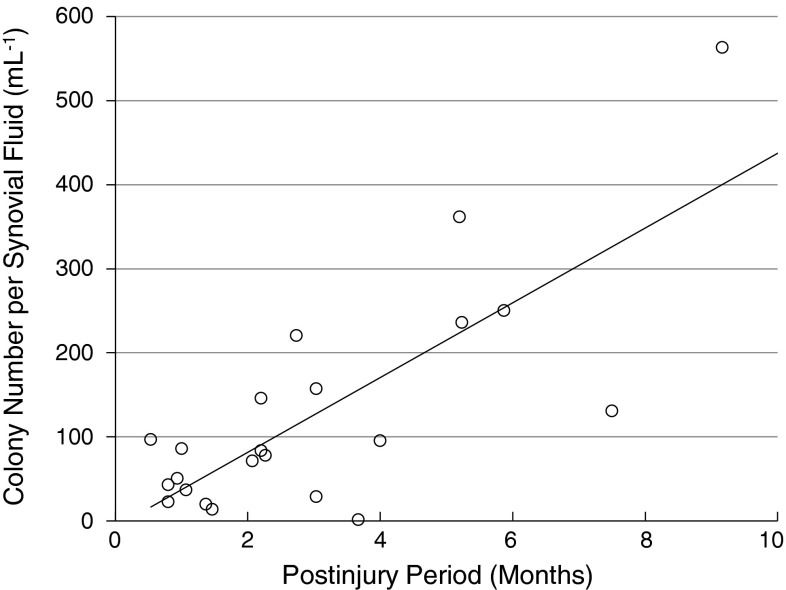
Colony number per synovial fluid (mL^−1^) was positively correlated with postinjury period (n = 22, r = 0.773, p < 0.001).

More colonies with traits of mesenchymal stem cells were observed in the knees with meniscus injuries than in the uninjured knees. The total colony number per synovial fluid was 253 ± 262 (mean ± SD) mL^−1^ in the meniscus injury group and 0.5 ± 0.9 (mean ± SD) mL^−1^ in the control group (Fig. 5). The number of mesenchymal stem cells per synovial fluid was 350 ± 370 x 10^3^ (mean ± SD) mL^−1^ in the meniscus injury group and 3.9 ± 6.7 x 10^3^ (mean ± SD) mL^−1^ in the control group. The number of colonies and total mesenchymal stem cells per synovial fluid were higher in the meniscus injury group than in the control group (p < 0.001). Colony number per synovial fluid (mL^−1^) was positively correlated with the postinjury period (r = 0.773, p < 0.001) (Fig. 6). The longer time passed from the injury, the more colonies of mesenchymal stem cells were observed in synovial fluid.

## Discussion

Meniscal injuries are common and important causes of knee dysfunction. The meniscus has a limited ability to heal spontaneously in the avascular area. However, although relatively uncommon, spontaneous healing from a meniscus injury has been observed even within the avascular area. This may be the result of the existence of mesenchymal stem cells in synovial fluid. However, the degree to which these cells may or may not be present in the human knee after meniscus injury had not been determined. Therefore, we investigate whether mesenchymal stem cells existed in the synovial fluid of the knee after meniscus injury.

This study had several limitations. The first limitation relates to the small cell number available in synovial fluid from uninjured knees. We could not compare the colony-forming ability in the same density of nucleated cells in the two groups. Initial cell density may affect the colony-forming ratio, but in this study, it was difficult to incubate two populations of the cells at similar densities because there were very few cells in synovial fluid in the uninjured knees. Also, the colony-forming cells from uninjured knees could not be analyzed for multipotentiality and surface epitopes because of the same reason. The second limitation is related to surface markers we examined. The International Society for Cellular Therapy proposed a criteria to define human mesenchymal stem cells, describing them as positive for CD105, CD73, and CD90 and negative for CD45, CD34, CD14 or CD11b, CD79α or CD19, and HLA-DR [2]. In this study, we examined all positive markers recommended. However, we examined only two negative markers among five recommended negative markers. To save the effort and expense, we carefully selected two negative markers that we thought to be the most promising. The third limitation is related to heterogeneity. We demonstrated that mesenchymal stem cell number per synovial fluid was higher in the meniscus injury group, and the majority of the cells were identical to the definition of mesenchymal stem cells in colony-forming, surface epitopes and multipotentiality. However, mesenchymal stem cells we termed here might contain other cells including adherent hematopoietic cells and just fibroblasts, although their number would be quite low because they do not form cell colonies like mesenchymal stem cells.

In this study, we demonstrated that mesenchymal stem cells existed in the synovial fluid of knees with meniscus injury. The cells studied in this article adhered to the culture dish, were spindle-shaped, formed cell colonies, and differentiated into chondrocytes, adipocytes, and calcified tissue. Also, the cells expressed CD44, CD73, and CD90 at a high rate; CD105 at a moderate rate; and did not express CD34 or CD45. These are consistent with the definition of mesenchymal stem cells [2].

There have been several papers describing mesenchymal stem cells derived from synovial fluid in the knee after ACL injury [17], osteoarthritis [11, 12, 15, 22], and rheumatoid arthritis [12]. Jones et al. [12] observed mesenchymal stem cells in synovial fluid in knees with meniscus injury. They compared mesenchymal stem cells in synovial fluid between the osteoarthritis group and the nonosteoarthritis group. This nonosteoarthritis group included meniscal injuries diagnosed by arthroscopy for otherwise unexplained joint pain, although our study is the first report that focused on mesenchymal stem cells in the synovial fluid in knees solely with injury to the meniscus.

The number of mesenchymal stem cells in synovial fluid was higher in the knees with meniscus injury than in healthy knees and increased during the postinjury period. We propose two possible mechanisms, one at the early phase and one at the late phase after injury.

Early on, bleeding in the knee can trigger an increase in mesenchymal stem cells in the knee. When the meniscus is injured, vessels in and around the meniscus rupture at the vascular area and bleed; consequently, the synovial fluid contains blood for several days. Vessel injury and bleeding promote the expression of cytokines and chemokines and consequently recruit mesenchymal stem cells. Generally, within a few days after meniscus injury, synovial fluid is bloody and then becomes transparent thereafter. In this study, the shortest period from the onset of meniscus injury to the time of synovial fluid aspiration was 14 days and the average period was 90 days. Most synovial fluid appeared transparent, indicating that intraarticular bleeding can trigger an increase of mesenchymal stem cells in synovial fluid only at the early postinjury phase. Inflammation may affect mesenchymal stem cells in synovial fluid. In our study, the amount of synovial fluid aspirated was 3.2 mL on average, and the synovial fluid appeared largely transparent, indicating a low number of inflammatory cells. Also, blood tests for C-reactive protein and erythrocyte sedimentation rate were within the normal range in all patients at that time (data not shown). Inflammation may increase the number of mesenchymal stem cells in synovial fluid at the early phase but would not maintain the increase of mesenchymal stem cells in synovial fluid for a long-term period.

Later, uncertain cartilage degeneration along with meniscus injury may cause an increase of mesenchymal stem cells in synovial fluid. In this study, all injured menisci were indicated for operations as a result of instability and in some cases locking had occurred. Several meta-analyses have demonstrated that an unstable meniscus causes osteoarthritis [5, 6, 9]. In this study, patients with severe cartilage injury or osteoarthritis were excluded; however, cartilage may degenerate after meniscus injury. According to our earlier work, the number of mesenchymal stem cells in synovial fluid was directly correlated to cartilage degeneration, evaluated with arthroscopy in ACL-injured knees, and the number of mesenchymal stem cells in synovial fluid increased along with the radiological grading of osteoarthritis [22]. If synovial fluid could be collected sequentially from immediately after meniscus injury, the number of mesenchymal stem cells in synovial fluid may transiently increase at an early phase because of bleedings in the knee, decrease after withdrawal of bleeding, then continue to increase along with cartilage degeneration resulting from dysfunction of the meniscus.

We postulate that mesenchymal stem cells participate in tissue repair. We previously reported that intraarticular injection of synovium-derived mesenchymal stem cells enhanced meniscus healing and regeneration in rat [10] and rabbit models [8]. Therefore, mesenchymal stem cells appear to promote meniscal healing. However, there may be too few mesenchymal stem cells in synovial fluid for menisci to heal spontaneously in most cases. We speculate that if mesenchymal stem cells in synovial fluid could be increased through an intervention, this may promote meniscus healing. This will call for future study.

In conclusion, we found that mesenchymal stem cells exist in synovial fluid in knees with meniscus injury. More mesenchymal stem cells were present in the synovial fluid of knees with meniscus injury than in normal knees. The number of colonies from synovial fluid was positively correlated with the postinjury period. Mesenchymal stem cells in synovial fluid may have a function to enhance spontaneous meniscal healing. Therefore, in future studies we will seek to clarify a mechanism for increasing mesenchymal stem cells in synovial fluid after injury, and we will try to determine whether this results in increased healing of meniscal tears.
